# Does pay for performance promote inverse inequality in chronic disease management?

**DOI:** 10.1093/fampra/cmaf025

**Published:** 2025-05-12

**Authors:** Sarah Linnane, Sarah Mullarkey, Eoin Kyne, Maeve Healy, John Fallon, Santosh Sharma, Ailish Hannigan, Andrew O’Regan, Ray O’Connor

**Affiliations:** Irish College of General Practitioners Mid-West GP Training Scheme, School of Medicine Building, University of Limerick, Plassey, Limerick V94T9PX, Ireland; Irish College of General Practitioners Mid-West GP Training Scheme, School of Medicine Building, University of Limerick, Plassey, Limerick V94T9PX, Ireland; Irish College of General Practitioners Mid-West GP Training Scheme, School of Medicine Building, University of Limerick, Plassey, Limerick V94T9PX, Ireland; Irish College of General Practitioners Mid-West GP Training Scheme, School of Medicine Building, University of Limerick, Plassey, Limerick V94T9PX, Ireland; Irish College of General Practitioners Mid-West GP Training Scheme, School of Medicine Building, University of Limerick, Plassey, Limerick V94T9PX, Ireland; School of Medicine, Faculty of Education and Health Sciences, University of Limerick, Plassey, Limerick V94T9PX, Ireland; School of Medicine, Faculty of Education and Health Sciences, University of Limerick, Plassey, Limerick V94T9PX, Ireland; School of Medicine, Faculty of Education and Health Sciences, University of Limerick, Plassey, Limerick V94T9PX, Ireland; Irish College of General Practitioners Mid-West GP Training Scheme, School of Medicine Building, University of Limerick, Plassey, Limerick V94T9PX, Ireland; School of Medicine, Faculty of Education and Health Sciences, University of Limerick, Plassey, Limerick V94T9PX, Ireland

**Keywords:** chronic disease, primary health care, health inequities, pay for performance, quality of health care, access to care

## Abstract

**Background:**

In Ireland, a mixed public-private system exists, whereby some patients receive state-funded general practice (GP) care under the General Medical Services (GMS), while private patients (PPs) pay fees. In 2020, the chronic disease management programme was introduced at the practice level to enhance the management of eight conditions. This pay for performance (P4P) programme incentivises GPs to review GMS patients regularly using a structured protocol. It is hypothesized that ineligible PPs receiving ‘routine care’, receive a poorer standard of care.

**Objective:**

To investigate the effect of P4P on the standard of care between PPs and GMS patients.

**Methods:**

Retrospective cross-sectional study involving 11 GP practices in the Midwest of Ireland. Clinical parameters recorded for the previous 12 months on 25 GMS patients and 25 PPs, matched by age group, sex, and one clinical condition, were collected from each practice. Parameters included vaccination status, and recording of: blood pressure, smoking status, renal function, glycosylated haemoglobin, and lipids.

**Results:**

Data from 550 patients showed that GMS patients were more likely than PPs to have received/been offered vaccinations (influenza (66% vs 26%), COVID-19 (69% vs 23%), pneumococcal (59% vs 15%)). GMS patients were more likely than PPs to have other parameters measured: blood pressure (92% vs 54%); smoking status (84% vs 24%); renal function (90% vs 59%); glycated haemoglobin (87% vs 56%); lipids (89% vs 57%) (*P* < .001 for all parameters).

**Conclusion:**

Significant disparities exist in the management of chronic disease in Ireland between GMS patients and PPs. Limiting P4P programmes to GMS patients promotes inequality.

**Key messages**

- Ireland launched the chronic disease management programme in 2020.
- This pay-for-performance (P4P) scheme is available to qualifying public patients.
- Study found superior standard of disease monitoring for qualifying patients.
- Private patients, excluded from the P4P scheme, were monitored less frequently.
- Study highlights the need for equitable P4P design to reduce inequality.

## Background

Three out of four deaths worldwide are caused by chronic disease, equivalent to 41 million people dying each year [1]. Furthermore, the prevalence of chronic disease and the associated economic burden will rise with ageing population demographics [2]. Health behaviours, including smoking, alcohol, physical inactivity, and diet, have long been identified as key modifiable risk factors for disease outcomes [3]. The role of primary care in addressing lifestyle as well as early diagnosis and management of chronic conditions has been recognized [4]. Chronic medical conditions are increasingly being referred to as non-communicable diseases (NCDs). However, because the financial support programme we discuss in this paper has been labelled the chronic disease management (CDM) programme, we will refer to these conditions throughout the paper as chronic diseases rather than NCDs.

To optimize CDM, many countries have introduced P4P programmes [5]. These structured programmes ensure patients receive standardized chronic disease care in general practice (GP) and practices are then rewarded for their ability to deliver defined outcomes. The programmes align remuneration with healthcare objectives related to quality, coordination, health improvement, and efficiency by rewarding the achievement of predetermined performance measures [5, 6]. P4P programmes can promote cost-effective use of resources [7]. Existing programmes are heterogenous in design and show varying levels of effectiveness, from absent to strongly beneficial [8, 9]. Effects on long-term disease outcomes are unclear [10, 11].

In 2004, the UK introduced the world’s largest P4P programme, the Quality and Outcomes Framework (QOF) [12]. While QOF indicated some improvements in care, it was difficult to infer causality [12]. Following its introduction, there was a surge of improvement in some measures, but this subsequently plateaued [13, 14]. Recording of processes tends to be more efficient when incentivised, which may account for some of the initial observed surge in uptake [15]. QOF was noted to have a detrimental effect on non-incentivised conditions [14]. Removal of incentives has been associated with a decline in performance [16].

Ireland has a mixed public/private healthcare system. Eligibility for free GP care is mainly based on low income and age [17]. A minority are granted discretionary eligibility based on high medical expenses [17]. Twenty-nine percent of the population qualify for free GP care through the largely means-tested General Medical Services (GMS) scheme [18]. The GMS system in Ireland also allows for severity of illness as well as low income in accepting members, so GMS patients may have more severe illness than private patients (PPs).

Ten percent of the population qualify for a doctor visit card, including those aged over 70 years and under 8 years, entitling them to free GP care irrespective of financial status [18, 19]. For clarity, we will refer to everyone eligible for free GP care as GMS patients and those ineligible as PPs. In 2020, the CDM P4P programme was introduced to enhance the management of eight chronic diseases, including asthma, chronic obstructive pulmonary disease (COPD), atrial fibrillation, stroke, transient ischaemic attack (TIA), type 2 diabetes mellitus (T2DM), ischaemic heart disease (IHD), and heart failure (HF). Incentivised GPs review GMS patients twice yearly according to a structured protocol. Now widely adopted by GPs, there is concern that ineligible PPs are receiving a comparatively poorer standard of chronic disease care [20]. A large proportion of PPs may be deterred by the financial cost of GP visits, investigations, or treatments [21].

The aim of this study was to assess the impact of the CDM programme on the management of matched GMS patients and PPs diagnosed with the named eight chronic conditions. The effect of this P4P programme would be clear because the PPs and GMS patients attend the same GP practices and differ only by their eligibility for free care with all care of all patients recorded on standardized electronic medical records (EMRs).

## Methods

A multi-practice retrospective cross-sectional study was conducted in the Mid-west region of Ireland, during the 12-month period from 1 July 2022 to 30 June 2023. Ethical approval was granted by the Irish College of General Practitioners Research Ethics Committee (ICGP_REC_2023_012).

### Participants and recruitment

All GP registrars on the Midwest GP training scheme were invited to participate in the study with written consent from their GP Principals. All practices participated in the CDM programme. Each registrar conducted a search using the coding system of the practice’s EMR software.

This software was used to generate patient lists for each condition and, using a random selection function, 25 GMS patients and 25 PPs, by age group, sex, and one clinical condition were selected in each practice. The patients must have attended the practice for at least 2 years prior to the start of the search period, ensuring adequate time for participation in the CDM programme.

### Data extraction

The data collection tool was designed by the research team and refined after initial piloting. Patient demographic data was recorded, including GMS/PP status, age under/over 70 years, and sex. The chronic disease(s) relevant to each patient was also recorded. Multimorbidity was recorded as the presence of more than one chronic condition. Each EMR was searched for the following processes of care: vaccination status (influenza, pneumococcal, COVID-19); blood pressure (BP); smoking status; renal function; glycosylated haemoglobin (HbA1c); lipid profile; brain natriuretic peptide (BNP) in patients with HF; and pulmonary function test in patients with COPD or asthma. Vaccination status was recorded as *given* if the vaccine was administered or if the patient was offered the vaccine but declined.

Aggregate anonymised data was entered into a password-protected spreadsheet and stored on the practice computer server until the completed file was then emailed securely to the principal investigator. Files were merged across all participating practices.

### Supplementary data

Following initial data collection, the authors investigated whether PPs were receiving chronic disease care in alternative clinical settings. Supplementary data was collected from external clinical letters within the patient EMR for a subgroup of PPs looking at previously mentioned processes of care.

### Data analysis

Mean with standard deviation was used to summarize normally distributed numeric variables. Median with interquartile range was used to summarize skewed numeric variables. Frequencies and percentages were used to summarize categorical variables. Chi-square test, Fisher’s exact test, two-sample *t*-test for means, and Mann-Whitney test for medians were used to compare characteristics and processes of care across the two groups (GMS and PP). A 5% level of significance was used for all tests with no adjustment for multiple testing. Due to the high prevalence of T2DM in the population, for this subset, GMS patients, and PPs were matched by age group and sex, using a propensity score matching technique. Multilevel mixed-effect logistic regression models were used for the process of care outcomes (care received yes or no) with fixed effects of age, sex, and group (GMS, PPs) and accounting for practice-level variation with practice as a random effect. Adjusted odds ratios with 95% confidence intervals are reported. Variability in processes of care outcomes attributable to differences between practices was measured using the variance partitioning coefficient (VPC). All statistical analysis was undertaken using SPSS for Windows Version 29.

## Results

**Figure 1.**
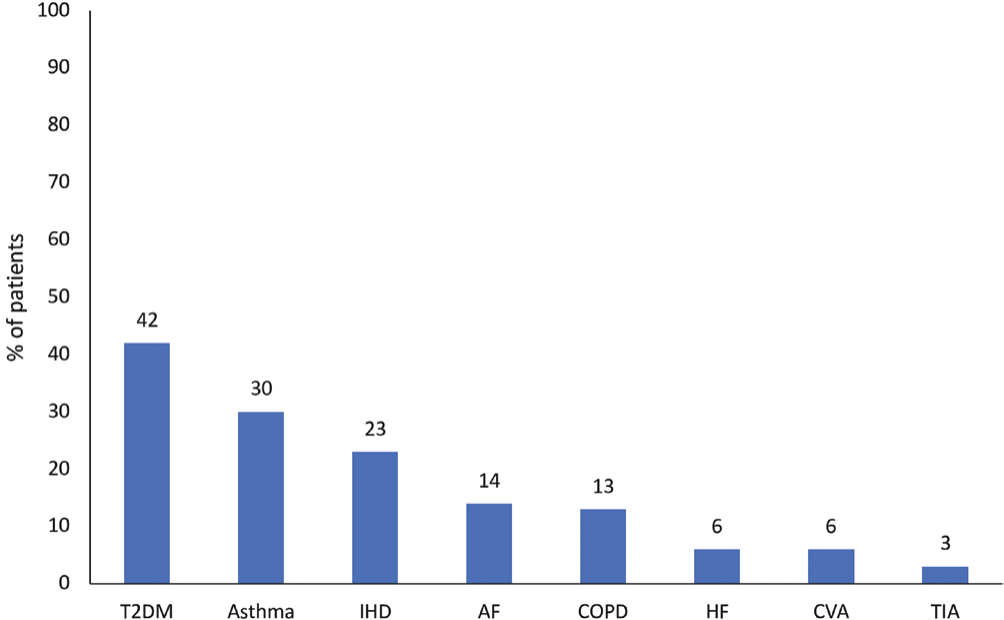
The prevalence of eight chronic diseases in the sample of 550 patients.

Eleven GP practices participated in the study, yielding data on 550 patients, evenly distributed between GMS and PP groups. The characteristics of the participating practices were similar to a nationally representative sample, in terms of size, workforce, and rurality [22] (Supplementary Table 1). The majority of patients were male (63%) and aged under 70 years (87%). The most common chronic diseases were: T2DM (*n* = 230; 42%), asthma (*n* = 165; 30%), and IHD (*n* = 125; 23%) (Fig. 1 and Supplementary Table 2).

The rate of chronic diseases in each group is shown in Supplementary Table 2. The rates in the GMS and PP groups were similar except for IHD (27% vs 18%, *P* = 0.013) and COPD (16% vs 10%, *P* = 0.035). GMS patients were more likely to have these conditions. Multimorbidity was also more common among GMS patients with 38% found to have more than one chronic condition, compared with 16% of PPs (*P* < 0.001).

**Figure 2.**
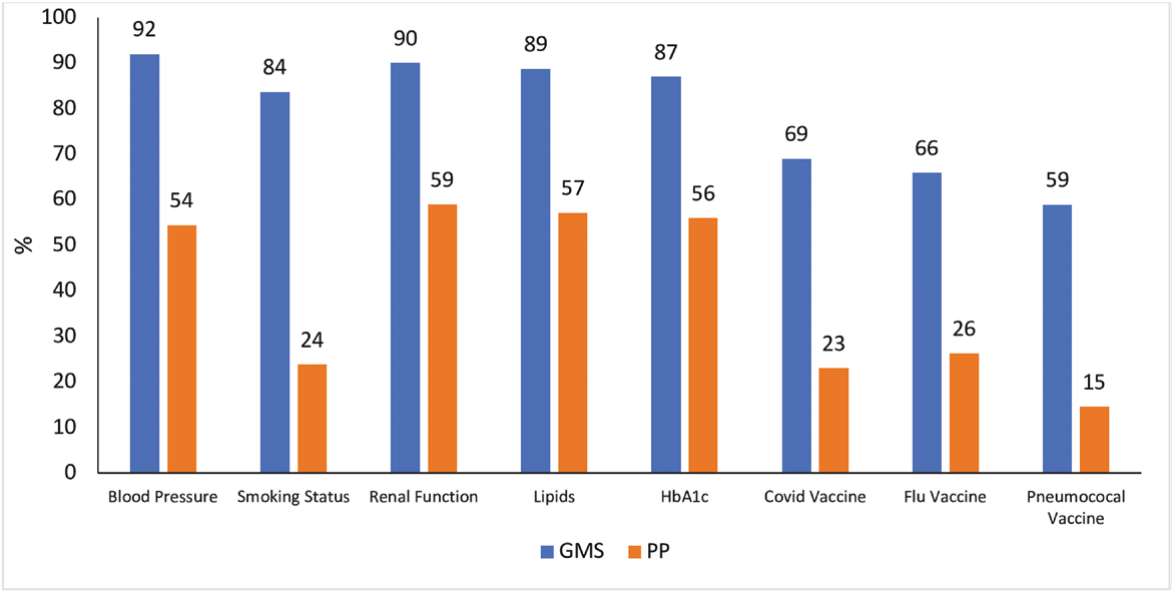
Comparison of percentages of GMS and PPs groups per processes of care parameters (*n* = 550).

The processes of care carried out for all patients are shown in Fig. 2 and Supplementary Table 3. Recording of these parameters was significantly higher for GMS patients compared to PPs (*P* < 0.001).

Disease-specific parameters, including BNP for those with HF (*n* = 32) and PFTs for those with asthma or COPD (*n* = 218), were recorded more frequently in GMS patients than in PPs (BNP: 66.7% vs 27.2%, PFTs: 18.2% vs 12.7%, respectively), although the difference was not statistically significant. (Supplementary Table 3).

**Figure 3.**
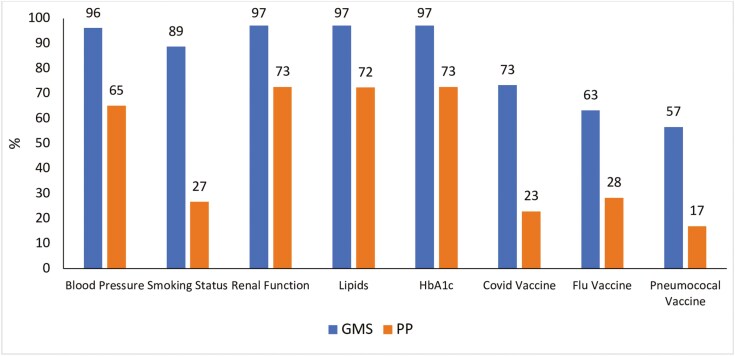
Comparison of percentages of GMS and PP patients within the T2DM cohort per processes of care (*n* = 212).

Subgroup analysis of the largest chronic disease cohort, patients with T2DM (*n* = 212) after matching by age group and sex, was carried out which showed similar statistically significant findings (*P* < 0.001) (Fig. 3).

**Figure 4.**
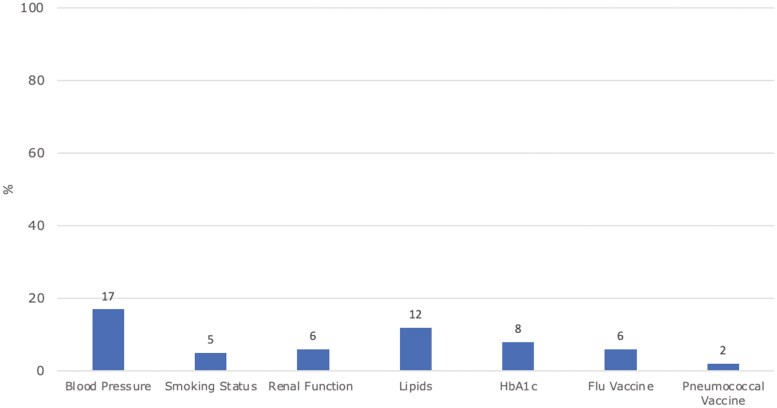
Results of PP supplementary analysis of processes of care documented outside the GP setting (*n* = 109). Note: COVID vaccine was not reviewed.

Figure 4 shows the results of supplementary data analysis on PPs (*n* = 109), undertaken to investigate if parameters were measured in another clinical setting. BP was most commonly documented (16.5%) followed by lipids (11.9%), but rates were low overall.

A multilevel logistic regression model was applied to understand the variation in processes of care. Patients aged 70 years and above had higher odds of being offered or given a COVID vaccine (adjusted odds ratio (AOR) = 1.87, 95% confidence interval (CI) 0.98, 3.58). There were no differences in the odds of receiving any of the processes of care between males and females. PPs had lower odds of receiving all processes of care compared to GMS patients (Table 1). Patients with multimorbidity had higher odds of being up to date with or offered the Pneumococcal vaccine (AOR = 1.90, 95% CI 1.13, 3.14), being given or offered the COVID vaccine (AOR = 1.69, 1.05, 2.72), and having their BP measured (AOR = 1.85, 95% CI 1.01, 3.37). The highest variability in processes of care attributable to differences between practices was found for the pneumococcal vaccine (VPC = 29.2%) followed by the Flu vaccine (VPC = 14.5%).

**Table 1.** Results of mixed-effect logistic regression for different processes of care (*n* = 550).

	Flu vaccine offered or given last 12 months	Pneumococcal vaccine offered or up to date	COVID vaccine offered or given in last 12 months	Blood pressure measured last 12 months	Lipid profile last 12 months
	Adjusted odds ratios (95% CI)				
Age group					
18–69	Reference	Reference	Reference	Reference	Reference
≥70	1.52 (0.82, 2.84)	2.00 (0.98, 4.06)	1.87 (0.98, 3.58)	0.65 (0.32, 1.33)	0.79 (0.41, 1.52)
Gender					
Male	Reference	Reference	Reference	Reference	Reference
Female	0.99 (0.65,1.51)	0.87 (0.54, 1.41)	0.90 (0.59, 1.38)	0.93 (0.59, 1.48)	1.18 (0.77, 1.83)
GMS/PP					
GMS	Reference	Reference	Reference	Reference	Reference
PP	0.17 (0.11, 0.25)	0.09 (0.05, 0.14)	0.13 (0.09, 0.20)	0.10 (0.06, 0.16)	0.18 (0.11, 0.28)
Number of chronic conditions					
One	Reference	Reference	Reference	Reference	Reference
Two or more	1.38 (0.88, 2.19)	1.90 (1.13, 3.14)	1.69 (1.05, 2.72)	1.85 (1.01, 3.37)	1.64 (0.95, 2.81)
VPC (%)	14.5	29.2	12.5	13.1	2.9

VPC: variance partition coefficients.

## Conclusions

### Summary

The aim of this study was to assess the impact of the P4P CDM programme on the management of matched GMS patients and PPs diagnosed with eight named chronic conditions. The first important and novel finding is that it results in healthcare process inequities between the GMS patients who are eligible for the programme and PPs who are not. The standard disease monitoring is superior among participating GMS patients. Statistically significant differences emerged in vaccination rates and the monitoring of health parameters including BP, smoking status, renal function, HbA1c, and lipids, with GMS patients consistently receiving more comprehensive care than PPs. Also, supplementary data collected from PPs’ records showed little evidence of chronic disease care being provided outside of the GP setting.

### Strengths

Strengths of this study include the fact that patient characteristics were similar except for eligibility for free GP care. All practices were GP training practices, which are likely to have higher standards than non-training practices due to specific accreditation requirements [23]. The data collection process was standardized, and data was gathered directly from the EMR.

### Limitations

Limitations include the relatively small sample size of 550 patients from a single geographical region. The majority of the sample were aged under 70 years which may limit generalisability of findings to older groups where chronic disease prevalence is higher. While GMS and PP groups had similar age groups and sex distributions, socio-economic status (SES) was not specifically measured. While most patients qualify for free GP care based on low income, others may qualify for other reasons such as age or carer status [19]. Eighty-seven percent of patients in this study were aged under 70 years suggesting the majority likely qualified based on low income [17].

It is possible that chronic disease care was provided in other settings such as another GP, pharmacy, or hospital. PPs of higher SES may be more likely to access care in private clinics.

Study outcomes were limited to processes of care and measurements, and did not assess long-term morbidity or mortality. GMS patients had more multimorbidity than PPs. Evidence suggests that patients with more chronic conditions visit their GP more frequently [24] and have longer consultations [25]; this could influence the results of this study.

### Comparison with existing literature

The finding that patients participating in the CDM programme have a more frequent recording of processes of care is consistent with other research [13]. A 2023 national report showed improvements in modifiable risk factors in those who attended three CDM appointments [20]. Although long-term disease outcome data is not yet available, the report shows that chronic disease is routinely managed in primary care rather than in the acute hospital setting [20]. This is in line with patients’ preferences [26], and has shown to be ideal for practical reasons [27].

The ‘inverse care law’ observes that those with greater medical needs have poorer access to medical care, and that this care is often of inferior quality [28]. Thus, people of lower SES generally have poorer health outcomes, including reduced life expectancy, than those in advantaged groups [29].

Several public health initiatives focussed on disadvantaged groups have been proposed to optimize health outcomes [30]. Lower SES, a known risk factor for chronic disease, can be targeted in its own right [29]. This study has shown that the CDM programme effectively targets the lower SES group of GMS patients, but in fact, they are now receiving a higher standard of care than PPs. Rather than narrowing the gap between socio-economic groups, the CDM programme has effectively addressed the inverse care law to an extent that places people ineligible at a serious disadvantage and risk.

Rose proposed that, since health risks are generally distributed in a continuum, a population approach could have better health outcomes than focussing on specific groups [30]. Childhood vaccination programmes are an example [31]. A study of incentivised maternity care provision in Rwanda [32]—a country with greater inequality than Ireland [33]—showed that, in the case of universally low service uptake, a non-targeted approach benefited all SES groups.

In 2018, the Irish Government introduced Sláintecare, a programme aiming to establish universal free healthcare. Continued implementation of CDM is intended [34], and the findings of this research support its future rollout to all relevant patients to improve equity.

The unintended consequences of P4P programmes have not been widely studied, but there is some evidence of negative effects on non-incentivised measures [8, 35]. We have shown that this can apply to excluded patient groups.

The design and implementation of P4P programmes are complex. Quality improvement and efficiencies are key goals [6], but few P4P programmes are designed to limit or reduce inequity [36] despite evidence that they should do so [37]. Design features to reduce disparities have been described [38] although these typically focus on targeting high-risk groups rather than making care more inclusive. A Scottish retrospective study of CDM suggested that its P4P programme was contributing to widening health inequalities over time. Levels of non-engagement in this programme were highest in socioeconomically deprived areas and physicians were permitted to exclude these patients without affecting incentivised performance targets [39].

Evaluating the effectiveness of P4P programmes is important to address inequity. The evidence on P4P programmes is evolving and critical evaluation and adaptation in healthcare policy and practice are needed [40]. A longitudinal study on health outcomes related to a P4P programme in Brazil showed that inequalities related to SES were eliminated when financial payments were adjusted for socio-economic inequalities [41]. Our study goes further, showing ‘inverse inequality’ whereby those with eligibility for free care due to lower incomes do better.

### Implications for research and practice

This study highlights the importance of P4P design and ongoing evaluation to promote equality. With data now available on the successful uptake of the CDM programme in Ireland, a feasibility study may be appropriate to consider an extension to the PP population, ensuring clinical efficacy and cost-effectiveness.

While our current findings suggest a positive influence on care for eligible patients, potential detrimental effects on those deemed ineligible from such a programme should not be ignored [42]. Larger-scale research would be useful to confirm that the current findings are applicable outside of the geographical area studied. We propose that healthcare should be determined by the patient’s need rather than their income, to prioritize medical needs ensuring equitable care for all patients living with chronic disease.

Thus, we recommend that payment to the attending GP should be the same for PPs and GMS patients and should be based on the presence of chronic disease rather than the perceived ability of the patient to pay for their medical care.

Finally, stakeholder involvement can maximize the potential benefits of P4P programmes [8]. Incorporating patients’ perspectives—both GMS and PPs—into refinement of the CDM programme would improve understanding of attitudes, thus fostering a nuanced approach to programme evaluation and development. Qualitative research into GP perspectives on CDM, particularly equitable care, could also offer valuable insights. Ireland is uniquely positioned to have population-based GP EMRs, designed to be significantly standardized, and also to include a de facto ‘control group’ of people (PPs in this instance) who are ineligible for the CDM program, facilitating a comprehensive examination of its effects.

## Supplementary Material

cmaf025_suppl_Supplementary_Tables_1-3

## Data Availability

Authors will make data available on reasonable request.
