# Supplementary material for: Does pay for performance promote inverse inequality in chronic disease management?
Source: Fam Pract. 2025 May 12;42(3):cmaf025. doi: 10.1093/fampra/cmaf025 (PMC12067410; doi:10.1093/fampra/cmaf025)
Supplement: cmaf025_suppl_Supplementary_Tables_1-3 [file cmaf025_suppl_supplementary_tables_1-3.pdf]

Supplementary Table 1: Distribution of GP practice characteristics (n=11)

Factor	n (%)
**Area**	
Urban	5 (45%)
Rural	6 (55%)
**No. GPs**	
Single-handed	4 (36%)
Group	7 (64%)

Supplementary Table 2: Distribution of patient characteristics by GMS and PP status (n =550)

Factor	Total (n=550)	GMS (n=276)	PP (n=274)	p-value
**Age Group**				0.033
18-69	479 (87.1%)	232 (84.1%)	247 (90.1%)	
>=70	71 (12.9%)	44 (15.9%)	27 (9.9%)	
**Sex**				0.741
Male	345 (62.7%)	175 (63.4%)	170 (62.0%)	
Female	205 (37.3%)	101 (36.6%)	104 (38.0%)	
**T2DM**	230 (41.8%)	124 (44.9%)	106 (38.7%)	0.138
**Asthma**	165 (30.0%)	85 (30.8%)	80 (29.2%)	0.682
**COPD**	73 (13.3%)	45 (16.3%)	28 (10.2%)	0.035
**IHD**	125 (22.7%)	75 (27.2%)	50 (18.2%)	0.013
**CCF**	34 (6.2%)	23 (8.3%)	11 (4.0%)	0.035
**AF**	79 (14.4%)	45 (16.3%)	34 (12.4%)	0.224
**TIA**	15 (2.7%)	9 (3.3%)	6 (2.2%)	0.602
**CVA**	33 (6.0%)	21 (7.6%)	12 (4.4%)	0.15
**Number of chronic conditions**				<0.001
1	400 (72.7%)	171 (62.0%)	229 (83.6%)	
2	110 (20.0%)	70 (25.4%)	40 (14.6%)	
>=3	40 (7.3%)	35 (12.7%)	5 (1.8%)	

Supplementary Table 3: Processes of care by GMS and PP status (n=550)

Factor	Total (n= 550)	GMS (n=276)	PP (n=274)	p-value
**Flu vaccine offered or given in the last 12 months**				<0.001
Yes	254 (46.2%)	182 (65.9%)	72 (26.3%)	
No	296 (53.8%)	94 (34.1%)	202 (73.7%)	
**COVID vaccine offered or given in last 12 months**				<0.001
Yes	250 (45.9%)	188 (68.9%)	62 (23.0%)	
No	293 (54.0%)	85 (31.1%)	208 (77.0%)	
**Pneumococcal vaccine offered or up to date**				<0.001
Yes	202 (36.8%)	162 (58.9%)	40 (14.6%)	
No	347 (63.2%)	113 (41.1%)	234 (85.4%)	
**Smoking recorded in the last 12 months**				<0.001
Yes	296 (53.9%)	231 (83.7%)	65 (23.8%)	
No	253 (46.1%)	45 (16.3%)	208 (76.2%)	
**Blood pressure measured in the last 12 months**				<0.001
Yes	403 (73.3%)	254 (92%)	149 (54.4%)	
No	147 (26.7%)	22 (8.0%)	125 (45.6%)	
**HbA1c measured in the last 12 months**				<0.001
Yes	392 (71.3%)	240 (87%)	152 (55.5%)	
No	158 (28.7%)	36 (13.0%)	122 (44.5%)	
**Renal function measured in the last 12 months**				<0.001
Yes	409 (74.4%)	247 (89.5%)	162 (59.1%)	
No	141 (25.6%)	29 (10.5%)	112 (40.9%)	
**Lipid profile measured in the last 12 months***				<0.001
Yes	400 (72.9%)	244 (88.4%)	156 (56.9%)	
No	148 (27.0%)	31 (11.2%)	117 (42.7%)	
**BNP measured in CCF in the last 12 months**	n=32	n=21	n=11	0.062
0	15 (46.9%)	7(33.3%)	8 (72.7%)	
≥1	17 (53.1%)	14 (66.7%)	3 (27.2%)	
**Lung function test (any) in patients with asthma or COPD in the last 12 months**	n=218	n=116	n=102	0.35
Yes	34 (15.6%)	21 (18.1%)	13 (12.7%)	
No	184(84.4%)	95 (81.9%)	89 87.3%)	

*2 cases lipid profiles are missing
